# Evaluating natural capital performance of urban development through system dynamics: A case study from London

**DOI:** 10.1016/j.scitotenv.2022.153673

**Published:** 2022-02-04

**Authors:** Jimmy O’Keeffe, Irene Pluchinotta, Simon De Stercke, Caitlin Hinson, Pepe Puchol-Salort, Ana Mijic, Nici Zimmermann, Alexandra M. Collins

**Affiliations:** a Centre for Environmental Policy, Imperial College London, UK; b Science and Solutions for a Changing Planet DTP, Imperial College London, UK; c Institute for Environmental Design and Engineering, The Bartlett Faculty of the Built Environment, University College London, UK; d Department of Civil and Environmental Engineering, Imperial College London, London, UK; e School of History and Geography, Dublin City University, Ireland

**Keywords:** Natural space performance, Ecosystem services, System dynamics, Natural capital, London

## Abstract

Natural capital plays a central role in urban functioning, reducing flooding, mitigating urban heat island effects, reducing air pollution, and improving urban biodiversity through provision of habitat space. There is also evidence on the role played by blue and green space in improving physical and mental health, reducing the burden on the health care service. Yet from an urban planning and development view, natural capital may be considered a nice to have, but not essential element of urban design; taking up valuable space which could otherwise be used for traditional built environment uses. While urban natural capital is largely recognised as a positive element, its benefits are difficult to measure both in space and time, making its inclusion in urban (re)development difficult to justify. Here, using a London case study and information provided by key stakeholders, we present a system dynamics (SD) modelling framework to assess the natural capital performance of development and aid design evaluation. A headline indicator: Natural Space Performance, is used to evaluate the capacity of natural space to provide ecosystem services, providing a semi-quantitative measure of system wide impacts of change within a combined natural, built and social system. We demonstrate the capacity of the model to explore how combined or individual changes in development design can affect natural capital and the provision of ecosystem services, for example, biodiversity or flood risk. By evaluating natural capital and ecosystem services over time, greater justification for their inclusion in planning and development can be derived, providing support for increased blue and green space within cities, improving urban sustainability and enhancing quality of life. Furthermore, the application of a SD approach captures key interactions between variables over time, showing system evolution while highlighting intervention opportunities.

## Graphical abstract

**Graphical Abstract:**
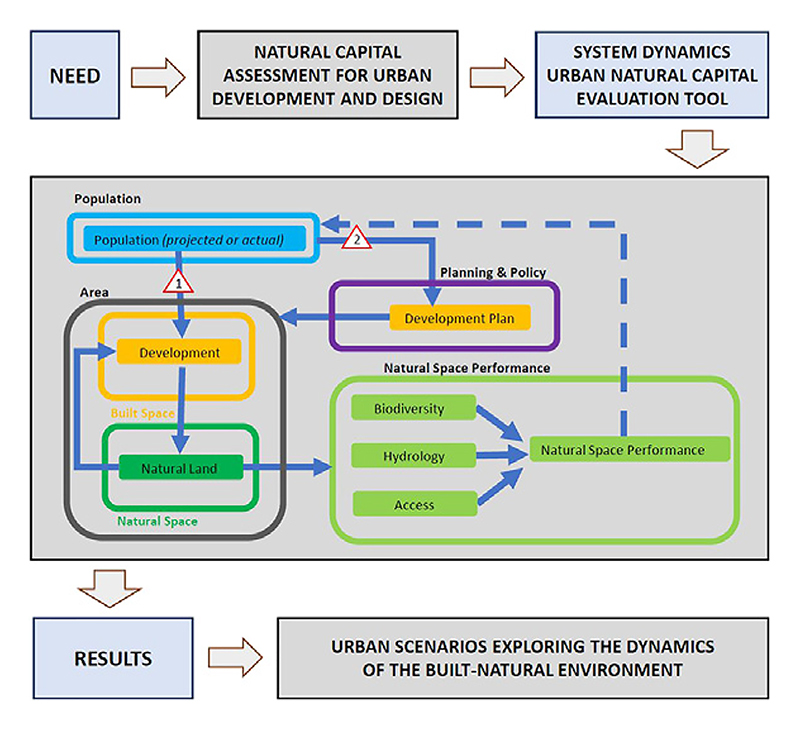

## 1 Introduction

Natural Capital (NC); the stocks of renewable and non-renewable natural resources that benefit people both directly and indirectly, and the flow of ecosystem services (ESS) these provide, have been increasingly recognised for their central role in sustaining economic and social wellbeing, societal resilience and sustainable development (Bateman and Mace, 2020; Guerry et al., 2015). As a result, NC has been incorporated into government policy processes (Department for Environment Food and Rural Affairs, 2020) and private sector decision making (Natural Capital Coalition, 2016; La Notte et al., 2017; Seppelt et al., 2011). For instance, it has been shown that NC and the flow of ESS significantly contribute to global and national gross domestic product (GDP) (Bradbury et al., 2021; Costanza et al., 2014) and underpin human wellbeing (Dasgupta, 2021). ESS are also produced by ecological structures within urban areas (Gutman, 2007; Jansson, 2013; McGranahan et al., 2005), and with over half of the Earth’s population now living in cities, and with rates of urbanisation increasing globally (United Nations, 2018), it is increasingly important to understand urban ESS; those that are either directly produced by ecological structures within urban or peri-urban areas, and the human-environment systems they depend upon (Bettencourt and West, 2010; Luederitz et al., 2015). Yet, there is a paucity of studies investigating ESS and NC generated in urban or peri-urban areas and a clear need for tools that can explain the value and benefits of nature in urban areas (Haase et al., 2014; Luederitz et al., 2015).

Unchecked and poorly planned urban development pose significant challenges to the provision of ESS. This is exacerbated by a lack of understanding of the inherent complexity within the built and natural environment system. In addition, ESS rarely conform to property, administrative or sectorial boundaries, leading to difficulties in management and regulation. However, Green Infrastructure (GI), the strategically planned network of natural or semi natural areas (European Commission, 2019) and Nature Based Solutions (NBS), approaches to help address societal challenges that involve working with and enhancing nature (Cohen-Sacham et al., 2016; European Commission (EC), 2015; Seddon et al., 2020), are increasingly seen as innovative solutions to transform NC into a source of green growth and sustainable development (Gómez Martín et al., 2020). While there is increasing support for GI and NBS in policy and planning documents, their actual implementation has been slow and examples of high quality NBS is the exception rather than the norm (see Fisher et al. (2020), Jerome et al. (2019) and Matthews et al. (2015)). There have been a number of reasons why this is the case including conflation of GI and NBS with traditional green spaces; the continued siloed approach to policy issues where NBS could play a role; a devaluing of NBS in local planning processes; a lack of consideration of long-term stewardship of GI and uncertainty as to what makes GI successful (Fisher et al., 2020). Yet, GI and NBS (used interchangeably in this paper) present a valid alternative to grey infrastructure for coping with climate-related risks in urban and rural areas alike (Calliari et al., 2019; Frantzeskaki, 2019; Giordano et al., 2019; Raymond et al., 2017). National, regional and local priorities in areas such as housing and highways often override environmental concerns (Pluchinotta et al., 2021b). Therefore, making explicit the long term societal and environmental contribution of NC is crucial to its implementation in urban design.

A comprehensive planning approach has the potential to harmonize human–environment interactions and mitigate the harmful impacts of urbanisation (Puchol-Salort et al., 2021). Such an approach requires planners and local decision makers to understand and value nature’s multiple contributions to the quality of urban life, through a whole systems approach. A recent study of decision makers in local authorities found a need for simple tools that allow incorporation of the importance of nature and green infrastructure into development projects (Pluchinotta et al., 2021b). Achieving this presents significant challenges, largely due to framework limitations and a lack of suitable headline indicators for assessing NC performance and measuring trade-offs between multiple interacting elements (Bateman and Mace, 2020). Emerging techniques that use outcome-based metrics and incremental management to progressively enhance ecosystem condition, and incorporate diverse stakeholders requirements and opinions across scales, sectors and knowledge systems, show promise, but are under-developed at present (Bateman and Mace, 2020).

A number of modelling platforms have been created to evaluate blue-green infrastructure and the outcomes of development on urban NC and ESS. These include the Natural Capital Planning Tool (NCPT) (Holzinger et al., 2019), InVEST (Hamel et al., 2021) and the Benefits Estimation tool (B£ST) (Horton et al., 2019). NCPT allows the impacts of new or proposed developments on NC or ESS to be assessed, with outputs described through a development impact score. InVEST is a suite of modelling tools designed to support city planning by providing information on natural infrastructure and the services it provides to people, complementing more comprehensive planning tools that capture built infrastructure and socio-economic dimensions (Hamel et al., 2021). B£ST was created to help assess and monetise the financial, social and environmental benefits of blue-green infrastructure, with a focus on sustainable urban drainage and natural flood management. Such models are extremely useful tools and help address critical gaps in evaluating NC and ESS, in some cases providing outputs which can be further utilised in other modelling frameworks. Yet, while they consider interactions between variables, the representation of system evolution over time is more challenging and results are predominantly provided in a pre- and post-development format, highlighting whether there is any net gain in assessed ESS from existing to new land uses. To fully understand built and natural environment systems and the implications of change, it is important to assess how they interact and evolve over time (Meadows and Wright, 2008). Doing so helps decision makers better understand the trajectory of change, identify tipping points and areas where timely interjections can improve outcomes while helping facilitate discussion among stakeholders.

System dynamics (SD) is an approach for conceptualising, analysing and understanding dynamic complex systems (Sterman, 2000). Based on closed chains of relations and feedbacks, SD modelling is well suited to representing the complexity of the integrated built and natural environment (Coletta et al., 2020; Giordano et al., 2019). It provides a useful tool for urban architects, planners, developers and decision makers to identify appropriate design and management strategies while helping policy makers develop sustainable approaches to urban planning (see Hall et al., 2013; Whyte et al., 2020). Using participatory modelling and Group Model Building SD is a well-known tool to allow participation in practice (e.g., Vennix et al., 1996). While SD modelling has been used to explore urban design challenges (e.g., Pluchinotta et al., 2021a), it is also uniquely suited to evaluate the ESS of a proposed or existing blue or green space. As SD modelling is based on integral equations to represent variables as stocks, and changes in these as flows (Eker et al., 2018), the methodology has potential to help improve the conceptualisation of NC and the influence land use planning decisions can have on it and the services it provides.

Through the use of group modelling exercises, SD modelling also facilitates a participatory approach (Eker et al., 2018), allowing the inclusion of stakeholders views (Pluchinotta et al., 2021a). Within environmental decision making the importance of stakeholder participation has long been recognised at the international and local level (Reed et al., 2009; UN, 1992; UNECE, 1998) and has been identified as essential for fair, sustainable (Gokhelashvili, 2015; Reed et al., 2009) high quality and durable decisions (Beierle, 2002; Fisher et al., 2009; Reed et al., 2009). However, participation is often identified as lacking within the NC and urban ESS approaches (Campbell-Arvai and Lindquist, 2021). If the urban ESS concept is to be a useful tool for sustainable urban planning, stakeholders’ perceptions of urban ESS should be considered more carefully in research (Luederitz et al., 2015). Additionally, participatory modelling can empower local stakeholders through the inclusion of their views, concerns and aspirations in the decision making process (Klain and Chan, 2012). In this paper, using stakeholder knowledge as a foundation, we develop a SD model representing urban development to explore the potential of the approach in articulating the impacts on and benefits of NC. The novelty of our work lies in using SD to understand and evaluate NC within a complex and changing human and natural environment. However, our primary goal is a dynamic representation of Urban Natural Capital (UNC), improving our understanding of the trade-offs and opportunities of socio-economic, built and natural environment change.

In Section 2 we provide a description of the study area; an urban space containing a significant amount of NC which is currently undergoing redevelopment. This is followed by an overview of the collaborative investigation of the Thamesmead area. Section 3 describes the translation of this knowledge into a SD model capable of evaluating the performance of natural space. The model is then applied to the study area in Section 4 where a number of different scenarios are explored to demonstrate the efficacy of the approach to support the understanding of how NC and ESS in urban environments may be affected by new development.

## 2 Materials and methods

### 2.1 The study area: Thamesmead Waterfront Development

**Fig. 1:**
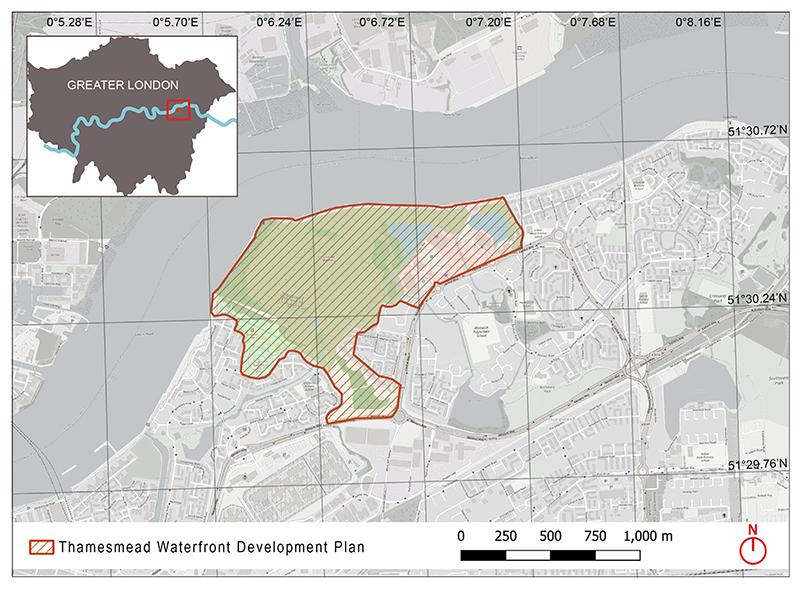
Thamesmead Waterfront Development area. The red line on the main map denotes the study site boundaries.

Originally built as part of the 1967 Greater London Council (GLC) Masterplan, Thamesmead is a 750 ha neighbourhood located in Southeast London. It sits between two London boroughs (Greenwich and Bexley) and is comprised predominantly of social housing, featuring post-war architecture (Cherry and Pevsner, 1983). Since completion, housing and infrastructure in the area has deteriorated significantly. A regeneration and development programme, led by the primary owners of the land; Peabody Housing Association, was initiated in 2014. The current population of Thamesmead is approximately 45,000 people in 16,000 housing units, approximately 6500 of which are owned by Peabody (Ford and Baikie, 2018). This number is expected to increase to approximately 100,000 residents by 2050 following redevelopment (Puchol-Salort et al., 2021). There are over 150 ha of blue and green space within the wider Thamesmead area, 65% of which is owned by Peabody (Askew, 2018; Puchol-Salort et al., 2021). It includes 32 ha of water bodies with several lakes and more than 7 km of canal network; 5 neighbourhood parks; and 14 Sites of Nature Conservation Interest (SNCI) (Askew, 2018). Recent studies estimate the potential NC value of the blue and green space of Thamesmead to be at least £306 million or £257 per resident per year (Askew, 2018; Vivid Economics, 2018). However, most of the blue and green space in the study area is underused by the residents with significant area currently inaccessible. The application of the model is based on the Thamesmead Waterfront Development; a new site which does not currently have a population (see Fig. 1).

### 2.2 Collaborative investigation of the Thamesmead blue-green and built environment

SD modelling processes can include both qualitative/conceptual and quantitative/numerical modelling phases (e.g. Pagano et al., 2019). Within the Thamesmead case study, a participatory qualitative modelling process was carried out to bring together *organizational and institutional stakeholders*, including developers, regulatory bodies, NGO’s and local Government, to jointly scope the focus of several quantitative SD models around the built and natural environment and sustainability. Specifically, the qualitative modelling phase aimed (i) to collaboratively identify a shared concern (namely a shared formulation of a “problem” which serves as a representation of the different concerns and stakes carried by the different stake-holders, see Ostanello and Tsoukiàs, 1993; Pluchinotta et al., 2019); (ii) to build a number of Causal Loop Diagrams (CLD) around the identified shared concern in order to gather knowledge on the system and to capture different perceptions from each stakeholder group. The identified shared concern was: how best to sustain and increase the quality of Built/Blue/Green space to ensure long term stewardship. Between 10 and 15 stakeholders participated in each workshop. A detailed description of the participatory qualitative modelling process is described in (Pluchinotta et al., 2021b).

**Fig. 2:**
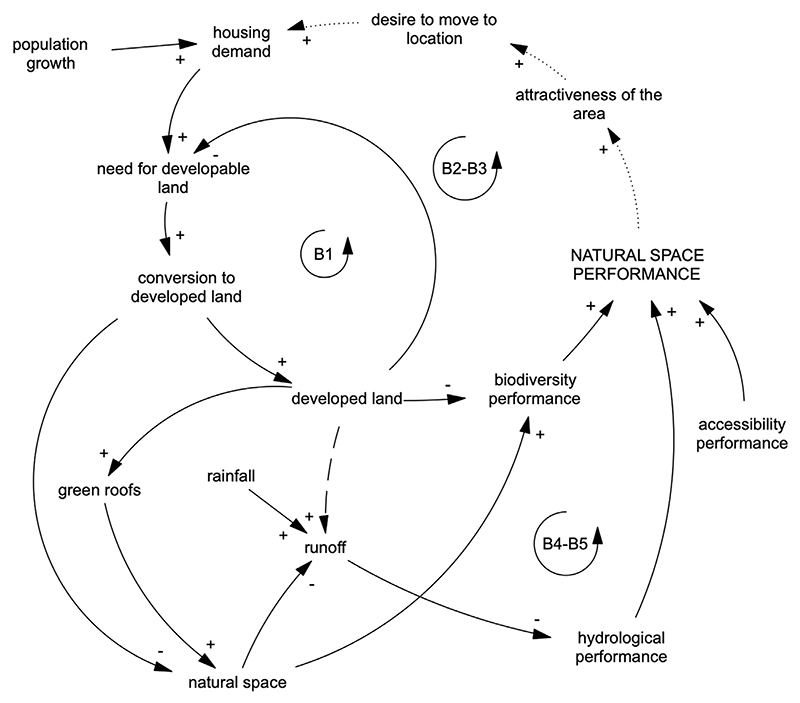
CLD to describe the connections and feedback between variables highlighted by case study stakeholders. Dashed lines refer (1) to the implicit connection between the natural space performance and housing demand via the attractiveness of the area and desire to live there; (2) the creation of runoff from built area which was not explicitly included in the current version of the model.

At the end of the participatory qualitative phase, as described in Pluchinotta et al. (2021b), stakeholders identified NC and Natural Space Performance (NSP) as one of the key issues and a priority to investigate, via a voting poll and a group discussion. Afterwards, several modelling sessions between academic experts were held for the creation of the CLD presented in Fig. 2 and the related SD model, which focused on changes in the quality of natural space from a NC viewpoint. The model can evaluate the capacity of natural space within the study area to provide ESS over time in relation to change.

The modellers developed the main structure, equations and parameter values of the SD model mainly using the information gathered from the scientific literature and technical reports (Please see Appendix A of this paper for further details). Experts in hydrology (5), NC (2) and urban environment (2) also shared their knowledge and supported the main modellers during the process. The modelling meetings were mainly between 1 or 2 experts and the modellers and focused on discussing and improving specific sectors of the model. Following the completion of the model prototype, an additional stakeholder workshop was held with key stakeholders (namely members of social-environmental NGOs working in the area with specific technical knowledge), during which the model structure and operation was described and validated. While participants suggested alternative equations, which could be used for variable change calculation, the overall structure of the framework was deemed to be accurate. The validation activity lasted two hours and involved a request for feedback on the overall structure of the model and focused on specific items of the model, for example, the type of land, the dynamics of change in developable land and the idea of the space performance indicator. In addition, during the validation activity, the stakeholders highlighted the usefulness of the framework, both for system understanding and information dissemination.

Therefore, the SD model presented was developed using both qualitative and quantitative information obtained through a combination of discussions with key stakeholders and subject experts. Information includes relevant variables for UNC and GI functioning in the study area, for example local population, the area of built and natural space, biodiversity, access to space and rainfall runoff.

A CLD describing the framework is presented in Fig. 2. The balancing loops B2 and B3, show how the ‘biodiversity performance’ and the related ‘natural space performance’ is highly dependent on both the quantity of ‘developed land’ and ‘natural space’. Similarly, B4 and B5 link the ‘hydrological performance’ with land type. The CLD includes an implicit link between ‘natural space performance’ and the desire to move to a particular area. The current version of the SD model does not include this aspect due to lack of robust data for parametrization. Moreover, the hydrological link between the built environment and run off was also included in the CLD for completeness, but not in the SD model, as this version was primarily focused on evaluating the performance of the natural space surrounding the development. This will be addressed in a future version as the model in its current form does not explicitly include the built environment. The model structure and simulated scenarios, along with references to relevant data sources are outlined in the following sections and in Appendix A: Model documentation.

## 3 A natural capital system dynamics model as evaluation framework for urban development

The model described in the following sections centres on the interconnections between the natural and human environment. Key variables and connections outlined by stakeholders and experts form the core of the developed model providing a generic framework capable of representing the impacts of change in many urban settings, including policy, management or design interventions, as well as social and environmental variations on the performance of natural space. This includes changes in population, climate and different management strategies and how these are likely to play out over a particular time frame. We describe the model rationale and equations in the following sections. However, the authors stress that while the model equations and parameters are appropriate in the context of the case study application, they can be changed and updated as needed to ensure the framework is relevant to each location. The aim of this adaptable framework is to provide a means of evaluating how portfolios comprising of different natural, built and social elements affect the provision of NC and therefore the sustainability of related decisions.

### 3.1 Model structure

**Fig. 3:**
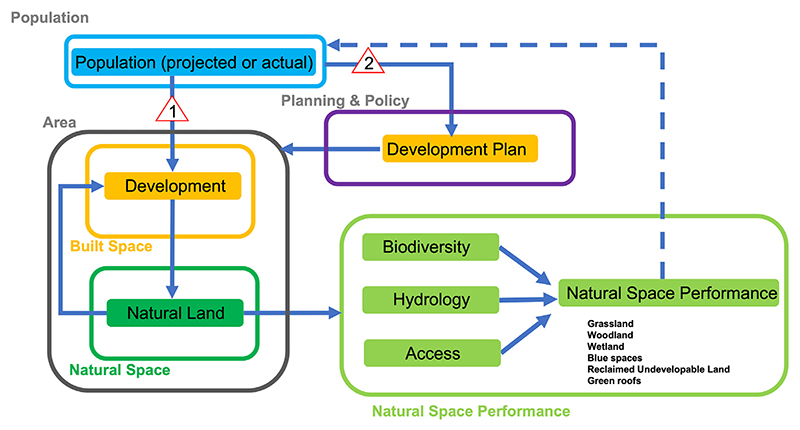
Schematic overview of the model highlighting key variables and how they are connected within the modelling framework. Natural Space Performance (NSP) is the headline indicator. Changes in Development can take place directly, through actual increases in population (Option 1), or indirectly through the creation of a development plan created to accommodate a projected population (Option 2). The dashed line between the natural space performance and the population is an implicit link, not currently represented in the model outputs.

**Fig. 4:**
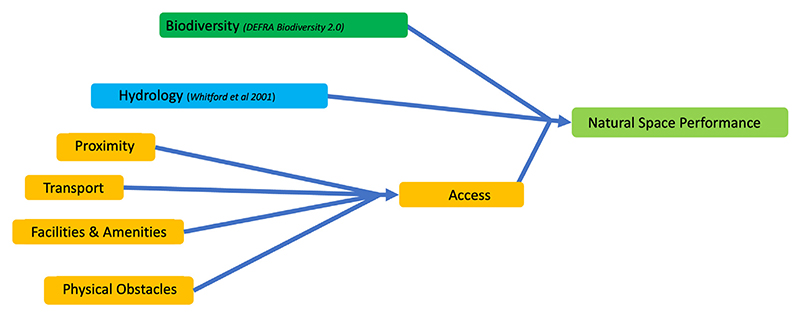
Representation of natural space performance for each individual land use type within the modelling framework. The order of calculations goes from left to right.

The participatory modelling process and subsequent refinement of the model resulted in the variables and associated connections described in the sections below and shown graphically in Figs. 2, 3 and 4. In order to minimise bias, the values for the baseline model run were set through inclusion of inputs from a wide range of stakeholders through an extended participatory modelling process that was carried out within the case study. The model runs on a timestep of 1/8th of a year in order to provide model stability and efficiency.

#### 3.1.1 Population (expected and actual)

Change in population is a primary driver of housing demand and represent one of the main reasons behind the Thamesmead Waterfront Development project. While this modelling framework has the capacity to directly model changes in population through the number of people moving into and out of an area, birth and death rates, we instead use the projected population (Askew, 2018), in order to explore the outcomes of planned and potential future scenarios. There are many factors which influence the number of people moving into and out of a location, including the quality of its blue and green space. Data shows that properties less than 100 m from green space are on average £2500 more expensive than those greater than 500 m from green spaces (Office for National Statistics, 2019). Desirability is also influenced by the quality of the natural space offer which includes the ecological quality and the condition of the space, its maintenance, safety and aesthetics. Therefore, the model accounts for the feedback between the performance of the natural space and its attractiveness and how that influences the number of people moving into and out of a location. However, in the current implementation of the model the latter is not used as an input; in other words, actual occupancy of the houses built does not feature.

#### 3.1.2 Planning and policy

While population and the subsequent need to provide accommodation are among the most dominant drivers for change in housing demand, individual developments are also influenced by local and national government policy, as well as development opportunities taken up by private developers. This may include large scale construction leading to the transformation of a brown or greenfield site into a new residential or commercial area. These large-scale development decisions can significantly and quickly impact the landscape and are separate and more impactful than gradual changes driven by individual or small-scale house building projects. This modelling framework can directly incorporate proposed or approved development plans including, for example, the number and size of housing units, and the area of land used and time scales. An example of the latter is the development cycle of housing construction, which in the Thamesmead case study has a period of 5 years. Individual housing unit types and their characteristics, including area, number of floors and at what stage during the modelling run they will be constructed, are explicitly included in the model.

#### 3.1.3 Built space

This is the area of land in hectares either available for development (Developable Land) or has already been converted to developed land (Built Area). An initial developable land value is set at the beginning of the model run based on-site specific information. During the model run, this value can increase by re-zoning land from the other land use types, predominantly natural space, though the model can also consider the impracticality of developing some types of land, for example blue space or wetlands. The parameters for which this occurs are set by the modeller and depend on local policy and development plans. Changes in the amount of developable land is driven by the demand; typically, through an increase in population and the need to develop housing and associated infrastructure. The type of development depends on the local needs and characteristics.

#### 3.1.4 Natural space

We designate Natural Space as all land types which are not part of the built or developable land. It may also include reclaimed undevelopable land, which cannot be developed on but can also provide ESS. In our study area this represents a reclaimed landfill with the capacity to provide additional natural space for residents. Within the modelling framework the natural space area can be reduced and converted into Developable Land and Built Area, representing the spatial impacts of development on natural space area. Each natural land type, including grassland, woodland, wetland and bluespace, is treated separately. Their areas are converted into developable land area in an order and at a rate of change determined by the modeller, with inputs based on development plans. In the current model setup, once the initial developable land stock is exhausted, grassland is converted to developable land and built upon, followed by woodland. Marsh & Wetland, Blue-Space and Reclaimed Undevelopable Land are not impacted by development in this model application; however, this can be changed by the model user. The thresholds for land use, defined as the limits which an area of a land type can be reduced to, are also set by the modeller, and can be based on existing or proposed land use plans.

#### 3.1.5 Natural space variables

##### 3.1.5.1 Biodiversity

The natural space biodiversity performance is assessed using the Biodiversity Metric 2.0 (Natural England et al., 2019), which provides a value in the form of biodiversity units (BU) based on the following equation:

(1) $$\begin{array}{l} \;\text{Area}*\text{Distinctiveness}*\text{Condition}*\text{Strategic Location}*\text{Connectivity}\;\\ \;=\;\text{Biodiversity Units}\; \end{array}$$

In the model the calculated BU are compared to the maximum BU to provide a metric for biodiversity where 0 is the lowest quality and 1 is the highest. Data describing the condition of the habitat within the natural area is normally collected through a mixture of available surveys, and site visits. In the absence of collected field information, suitable values from literature can be used. A full description of the approach is outlined in the Biodiversity Metric 2.0 documentation (Natural England et al., 2019). In this application, due to a lack of suitable field derived data, we use expert opinion to approximate values for Condition, Strategic Location and Connectivity. Area for each land use type is outlined in Appendix A. This calculation is undertaken individually for each land use type.

#### 3.1.6 Hydrological performance

A significant impact of urbanisation is the change in the natural water flow regime. This is most evident during urban flooding events, where percolation of water to aquifers is prevented by built impermeable surfaces. These surfaces also speed up the overland flow of water to rivers leading to increased fluvial flooding. Flooding has been further exacerbated by an increased number of extreme rainfall events due to changes in climate. This is accounted for within the framework by including a hydrological performance metric.

Based on a methodology described by Whitford et al. (2001), the approach accounts for land use type, its permeability and generation of run-off:

(2) $$P_{e}=\frac{{\left(P-0.2S\right)}^{2}}{P+0.8S}$$

Where *Pe* is run off, *P* is precipitation (the model uses average annual rainfall), *S* is the maximum retention of the area where the greater the *S* value, the smaller the run-off. The value of *S* in mm, is given by:

(3) $$S=\frac{2540}{CN}-25.4$$

Where *CN* is the curve number which describes the land type and conditions. Fully impermeable and water surfaces have a *CN* value of 100. In this case *S* will be 0, whereas when the *CN* < 100, *S* will be positive. The curve number values in this application are taken from the USDA Hydrologic Soil-Cover Complexes (2004) and expert judgment. However, the practical design values of land use types validated by experience lie in the range of 40 to 98 (Deshmukh et al., 2013; Van Mullem, 1989). This results in a value for run off generated over the course of the year which is compared to the precipitation and converted into an indicator 1-P_e_/P. An area-weighted average of these values for the different land types is then normalised by the best-performing land type. This then becomes an overall performance metric. The value of which can range from 0 to 1, where 0 is poor performance likely to result in flooding and 1 is excellent performance less likely to result in flooding. This calculation is undertaken individually for each land use type.

#### 3.1.7 Access

Here defined as the physical and social capacity to engage with blue and green space, accessibility forms a central component in the provision of societal benefits from urban space. There is a growing body of evidence to support that access can play an important role in improving and maintaining mental and physical health (van den Berg et al., 2015; Gascon et al., 2016; Office of National Statistics and Public Health England, 2020). It has been shown that this accessibility is particularly important for more vulnerable socio-economic and minority ethnic groups who derive a disproportionately high benefit from parks and green space, despite being less likely to live close to them (Mayor of London, 2020). The variables highlighted as most important by key stakeholders and experts include physical obstacles, proximity, transport, and the availability of facilities. These were also outlined in Public Health England’s Improving access to greenspace (Office of National Statistics and Public Health England, 2020). Accessibility is the mean of proximity, transport, facilities and amenities and physical obstacles which are described in the following sections and in the Appendix 1 section on Natural Space Performance.

##### 3.1.7.1 Proximity

The distance of natural space from the homes of potential users is a major determinant of use; two thirds of visits to green space in the UK occur within two miles of the home (Office of National Statistics, Natural England, 2018). For the purposes of this model, we define proximity as the distance of green space from the home, following the approach used by Office of National Statistics and Public Health England (2020). This is reflected in the model through a user assigned value between 0.1 and 1, representing the average distance from the home to natural space. Green or blue space located less than 2 min' walk from the home is deemed excellent and given a value of 1. A value of 0.1 denotes greenspace located a walk equal or greater than 40 min from the home; the average time it takes to walk two miles.

##### 3.1.7.2 Transport

Transport infrastructure is an additional variable which determines the usability of public natural space. Public transport includes buses and trains, the number of stops and how regular the service is. Private transport includes parking facilities for cars and bikes, their quality, safety and cost if relevant. This is captured in the model through a user assigned value between 0.1 and 1, where 0.1 represents a scenario with little to no public transport or parking options, and 1 where excellent transport facilities exist.

##### 3.1.7.3 Availability of facilities

Public amenities include the presence of toilets and washroom facilities, cafes, playgrounds and play areas, benches and seating areas. The presence of particular types of amenities are crucial to the use of space by certain groups of people and their absence will significantly reduce the likelihood of the space being used. It should also be noted that the type of amenities within a park are location specific – public space design will be different for a green space adjacent to a large city office block than a suburban area with young families with children. The quality of facilities is represented as a value between 0.1 and 1, where 0.1 is public space with little or no facilities, and 1 is the presence of excellent facilities. Values are derived by stakeholders and expert guidance.

##### 3.1.7.4 Absence of physical obstacles

Physical obstacles to the use of urban natural space can be natural or manufactured and may include vegetation, topography, lack of or poorly maintained pathways, limited lighting and the presence of areas which reduce the safety of potential natural space users. Values range from 0.1 to 1 where 0.1 is a space with few to no paths, and/or areas which are inaccessible or unsafe to potential users. Areas with excellent facilities such as fully accessible seating areas, toilets and cafes are given a value of up to 1. These input parameters are obtained through expert guidance and stakeholder consultation, considering the different users of the space; their needs and how physical obstacles may inhibit safe and regular use. This included access for wheelchair users. These input parameters will vary depending on the case study and are qualitative scores that stakeholders and experts have agreed upon.

#### 3.1.8 Natural Space Performance (NSP)

The assessment of natural resource and ecosystem service sustainability is critical to system understanding and a key part of evidence generation for improved decision making. A number of approaches already exist, for example, the UK’s NC Accounts which estimate exchange prices that are directly comparable to GDP. However, whether or not society is on a sustainable trajectory is best accounted for as the aggregate of all NC assets (Bateman and Mace, 2020). In order to fully assess the state of human-natural environment interactions it is important to consider both the stocks of natural assets and the flow of ESS, thereby including resource sustainability in decision making; a factor which can be missed using simple flow based assessments (Bateman and Mace, 2020). Almost all natural resources are limited in some way; if a decision is made to change the stock or flow of a natural resource or eco-system service, it can reduce the possibility of further utilisation, generating an opportunity cost that may or may not be known when decisions are made (Bateman and Mace, 2020).

Following Yun et al. (2017), we consider ESS as a portfolio of assets. The overall performance of the portfolio depends on the performance of the underlying assets which are subsequently influenced by their interactions. These changes in portfolio performance provide an attractive headline index for ecosystem based management, regardless of whether ecosystem wealth is ultimately included in a broader wealth index (Yun et al., 2017). In this paper we develop an evaluation approach where key variables, such as biodiversity, hydrology and access (as described throughout Section 3) are combined and normalised. These values provide metrics, or headline indicators, which can be used to measure the performance of part of (e.g., changes in green or blue space hydrological performance) or the entire (e.g. the urban natural space in the study area) system in relation to change. These metrics provide a useful indication of system behaviour; however, our primary goal is a dynamic representation of UNC, improving our understandings of the trade-offs and opportunities of socio-economic, built and natural environment change. Creating such a composite indicator, however, is not trivial, as the reduction from three dimensions to one necessarily involves choices to be made. We based our choices on a list of requirements consistent with our conceptualisation of the headline index; Natural Space Performance (NSP).

The indicator expresses performance on a scale from 0 (complete absence of any benefit) to 1 (the highest physically possible performance) and should obey the Anna Karenina principle (Diamond, 1997), where a value of 1 can only be achieved if performance in each dimension is highest, while the lowest performance in either dimension (corresponding to a value 0 of the corresponding indicator) would lead the NSP index of indicator to take on the value 0. A mathematical form which satisfies these requirements is a weighted geometric mean of three dimensionless indicators, each representing one aspect of natural space:

(4) $$NSP={\left(BP^{\alpha_{BP}}\cdot HP^{\alpha_{HP}}\cdot AP^{\alpha_{AP}}\right)}^{\frac{1}{\alpha_{BP}+a_{HP}+\alpha_{AP}}}$$

Where BP, HP and AP are unit-less and stand for the biodiversity, hydrological and access performance indicators, respectively. Parameter weighting is represented by α. These indicators are constructed based on requirements that are similar to those for NSP. For example, an extreme case where there is an absence of natural space will result in a value of zero with no benefits provided, while an entire area comprised of the best performing natural space type will result in a value of 1. Additionally, we require that the contributions of each natural space type be positively correlated with its relative surface area.

These requirements are satisfied if we construct each of the indicators as a ratio of the current state and the best possible state. The numerator of this ratio is the sum product of the performance of each natural space type and its surface area (except for biodiversity, for which the area is already included in the metric as expressed in biodiversity units (BU)). The denominator is the product of the total area and the per-unit-area performance of the natural space type for which it is highest.

These indicators are combined into a composite indicator, NSP. However, there are still two degrees of freedom – the weights α – which rest upon value judgments that only stakeholders can make, e.g. through determining relative degrees of importance in pairwise comparisons. While we have included in the model the capacity to consider weighting, the results we present are obtained from model runs with all weights identical, the implicit choice being that in this application, all three aspects are equally important. The weights can be changed by users of the model according to the relative importance they attach to the three aspects.

## 4 Model application

The developed model was applied to the Thamesmead Waterfront Development case study to assess the performance of natural space over a number of different scenarios. Scenarios were chosen to represent likely environmental and land use trajectories in the coming years to explore the impact of differing options for the development and the usefulness of the NC Evaluation framework. The scenarios are plausible alternative permutations of the development plan (Peabody, 2019) designed by the modellers and validated by urban development experts and stakeholders. Key variables are listed in Table 1.

### 4.1 Scenarios

#### 4.1.1 Scenario 1 (S1): approximation of proposed building design (Baseline)

In this scenario, we increase built space area through implementation of a development, broadly in line with the proposed urban design for the Thamesmead Waterfront Development plan. For the purposes of this scenario, no changes are made to the natural space parameters, allowing us to directly explore the impacts of development on NSP.

#### 4.1.2 Scenario 2 (S2): high density building design

In S2, the development footprint is reduced through implementation of high-rise buildings. Under this scenario no additional changes are made to the natural space.

#### 4.1.3 Scenario 3 (S3): low density building design

This scenario explores the model’s ability to investigate the impacts of urban sprawl on natural space performance. The overall built area footprint is increased, requiring a significant reduction in natural space area. No additional changes are made to the natural space.

#### 4.1.4 Scenario 4 (S4): proposed building design with green roofs

Here we explore how the implementation of green roofs can affect the overall NSP, providing additional ESS including biodiversity, improved hydrological performance and greater natural space access.

#### 4.1.5 Scenario 5 (S5): the use of Nature Based Solutions to reduce flood risk

S5 examines the role that NBS can play in addressing environmental challenges, in this case flooding. By changing the rainfall runoff curve number (see Section 3.1.6), which simulates the effect of different vegetation types (see USDA, 2004), we explore how a reduction in flood risk can be achieved across the site under temporal variations in precipitation.

#### 4.1.6 Scenario 6 (S6): Integrated scenario

A combined implementation of parameters outlined in S4 and S5 are used in S6 to explore how natural space performance can be improved through the provision of NBS’s to reduce flood risk, increase biodiversity and provide additional high quality green space for residents.

#### 4.2 Results

**Fig. 5:**
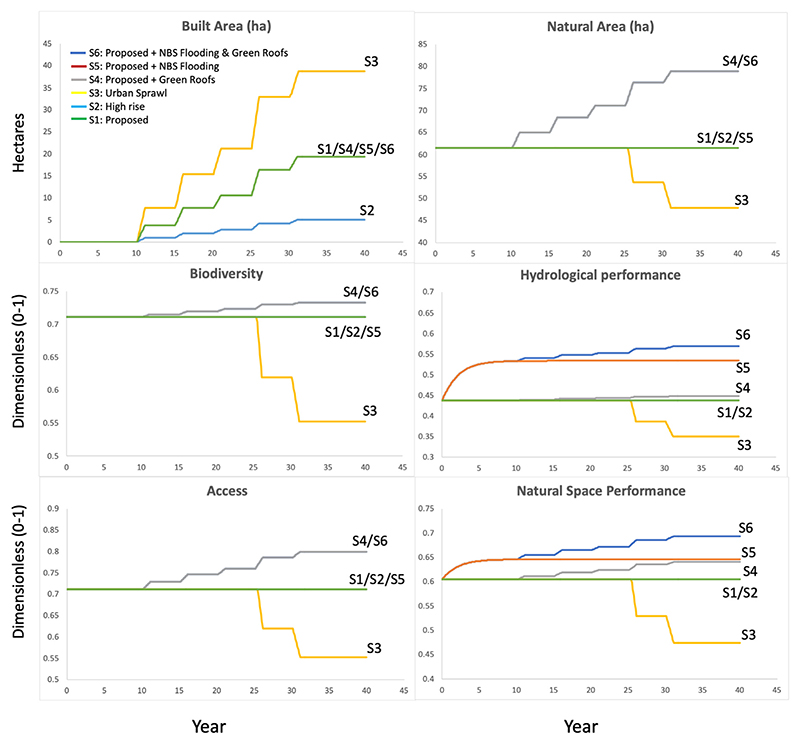
Outputs generated by the model during Scenarios 1–6. Results are presented by key variable. With the exceptions of Built Area and Natural Area which are presented in hectares (ha), outputs range from 0 to 1 and are dimensionless. Model outputs for some scenarios overlap (e.g. S2 & S5 underly S1 in the Biodiversity results).

The results of all scenarios are presented in Fig. 5. Many of the scenario outputs display results as a series of steps, for example, S3 in the Built Area plot. This represents the progression of construction through the building development plan which is scheduled to take part in stages. This information forms part of the model driving data (see Appendix A) and influences the rate of change that takes place during the model runs. Where model outputs reach a steady state, this represents the end of the development phase of the project. Changes in the natural or built environment, including the impact of maintenance are not considered as no suitable information was available. The following section describes model outputs by scenario.

#### 4.2.1 Scenario 1 (S1): approximation of proposed building design (Baseline)

S1 results in an increase of approximately 19 ha of built space taking place over 5 construction phases. No other changes occur to the key model values during the model run. As the area of the development in this scenario is less than that of the initial Developable Land (see Section 3.1.3), no reduction in natural space area takes place and it remains constant at 61 ha. Biodiversity values and hydrological performance also remain at 0.710 and 0.437 respectively, as no change in natural space area or to the relevant natural space variables are made. Under S1, accessibility and NSP remain constant at 0.711 at 0.604 respectively throughout the model run.

#### 4.2.2 Scenario 2 (S2): high density building design

S2 represents a higher density building scenario resulting in 5 ha of built space – the lowest of all explored potential scenarios. As with S1, the natural space area remains constant at 61 ha throughout the model run as under these scenarios the building configuration footprint does not lead to a reduction or increase in natural space. As a result, biodiversity values also remain constant at approximately 0.710, as does hydrological performance at 0.437 as no change to the relevant natural space variables are made. Under S2 accessibility values also remains constant at 0.711. Under S2, the only change made during the development is to housing design which reduces the building footprint and does not impact NSP. As a result, NSP remains constant at 0.604.

#### 4.2.3 Scenario 3 (S3): low density building design

S3 results in the largest development footprint; 39 ha of built space. This leads to a reduction in natural space, from 61 to 48 ha and the ESS it provides, including biodiversity values, which fall from 0.710 to 0.552. There is also a reduction in hydrological performance under S3, dropping from 0.437 to 0.349, due to an increase in impermeable area and a reduction in natural space areas and rainfall runoff retention capacity. Accessibility also falls during S3, from 0.711 to 0.552, due to the replacement of natural space area with private inaccessible property. The overall NSP during S3 reduces from 0.604 to 0.474.

#### 4.2.4 Scenario 4 (S4): proposed building design with green roofs

With the exception of green roof implementation, the configuration of building design under S4 is the same as that found in S1. Changes in land use under this scenario result in approximately 19 ha of built space, however, there is an increase in natural area, from 61.5 to 79.9 ha due to the addition of green roofs on buildings. This is reflected in biodiversity values, which increase from 0.710 to 0.733. The hydrological performance also increases during S4; from 0.437 to 0.472, as the presence of green roofs on buildings slightly improves the rainfall runoff capacity of the overall site. Access values during S4 also increase due to the addition of green roofs on buildings, from 0.711 to 0.799. While the implementation of green roofs in the construction design increased NSP during S4; from 0.604 to 0.640. It also improves the site’s capacity to provide ESS including a reduction in flood risk while offering additional, if limited, access to natural space.

#### 4.2.5 Scenario 5 (S5): the use of Nature Based Solutions to reduce flood risk

The S5 building configuration follows the same pattern as S1 and S4, resulting in approximately 19 ha of built space. Natural space area remains constant at 61 ha throughout the model run as no change is made to natural space area. Under S5, biodiversity units also remain constant at approximately 0.710 throughout the model run. The building configuration footprint does not lead to a reduction in natural space, however, the hydrological performance increases during S5 due to the gradual adaption of natural space to reduce flooding, increasing from 0.437 to 0.533. No other changes are made which affect hydrology. Natural space accessibility remains constant at 0.711 in this scenario. NSP values for S5 increase from 0.604 to 0.646. This is slightly higher than S1 and S4 due to the increased capacity to reduce flood risk.

#### 4.2.6 Scenario 6 (S6): integrated scenario

S6 follows a similar building configuration to S1, S4 and S5 resulting in approximately 19 ha of built space. The implementation of green roofs during S6 also sees an increase in natural area from 61.5 to 78.9 ha. This change supports an increase in biodiversity; from 0.710 to 0.733 and is also reflected in hydrological performance; increasing from 0.437 to 0.568 due to both green roof implementation and the adaptation of natural space to reduce rainfall runoff. Access values increase during S6 from 0.711 to 0.799. The implementation of green roofs as part of S6 leads to an increased capacity to provide ESS through additional natural area, leading to an increase in NSP from 0.604 to 0.693.

## 5 Discussion

This paper explores how SD can improve understanding of the complex built and natural environment, demonstrating, through a stakeholder informed SD model, its capacity to help planners, designers and developers reduce negative development impacts while maximising the provision of ESS from urban NC. A key benefit of using a SD model is its ability to explore the outcomes of a variety of design options, considering interaction and feedbacks between different key variables over time. Through a series of scenarios, we use the model to investigate the impacts of high- and low-density housing design, the implementation of green roofs and the use of NBS for flood risk reduction. These scenarios allow us to explore the dynamics of the urban human-natural environment, and how change in one element of design can propagate throughout the entire system. Despite a lack of qualitative and quantitative data, this model is well suited to NC evaluation and urban design, providing useful insights to study area development. This was supported by stakeholders who highlighted the usefulness of the framework, both for system understanding and information dissemination.

The S4 model outputs which describe the hydrological performance of the study area show that the addition of green roofs can play a role in the reduction of flood risk, though less than benefits which could occur by adaption of green space, through changes in vegetation type and corresponding CN for flood risk reduction (S5). However, focusing on just one ESS masks additional benefits which are provided. The inclusion of green roofs creates additional natural space, leading to improvements in biodiversity, and accessibility for residents. Here, the importance of taking a portfolio approach (Yun et al., 2017), where the overall result depends on the performance of the underlying assets and their interactions, becomes clear. The NSP of S4 is similar to that of S5, and while S5 benefits are as a result of targeted changes to improve hydrological performance, improvements in S4 are due to the increase in green space and the corresponding increases in biodiversity, accessibility and hydrological performance. It also supports the idea that we should not focus on improving a single ESS or addressing a single challenge. Without adequate evaluation, NBS are likely to appear less cost effective than traditional grey infrastructure and therefore less attractive to developers and planners (Mell et al., 2013). However, the model and results highlight how NC provides multiple benefits, including reduced flood risk and increased biodiversity, which should also be considered when designing or adapting urban space.

Globally, urban flooding is a growing problem (Fiori and Volpi, 2020), exacerbated by an increase in impermeable area which reduces the natural storage capacity and retention abilities of land to slow the movement of water to rivers and other water bodies. This is particularly evident during extreme rainfall events which are becoming more common under climate change (Li et al., 2020). Flood risk was highlighted as a concern among stakeholders and in the context of climate change, the ability of the development to deal with more frequent extreme rainfall events is required. Both S5 and S6 highlight the positive implication of NBS for flood risk reduction, helping balance the impact of development and the increase in impermeable area, as seen in S1 and S3. While developers may have limited capacity to implement change, the model allows the user to explore how different portions of land can be adapted to test the benefits of NFM, providing evidence on NBS which can be compared to traditional grey infrastructure. This approach helps highlight the potential operational and cost effectiveness of nature based solutions to urban challenges (Dick et al., 2019), helping reduce the strain on traditional drainage infrastructure while also providing additional benefits.

This paper shows, SD modelling can be used to effectively conceptualise NC, land use planning decisions and the ESS provided. To date, tools for the evaluation of NC have tended to look at pre and post build without considering the integrated nature of variability that takes place as urban development progresses (e.g., multi-criteria decision making tool, see Belton and Stewart, 2002; Mardani et al., 2015) which can assess impacts on multiple areas of concern but do not consider interactions or temporal changes. Using a SD approach allows for the consideration of development plans and complex scenarios over different spatial and temporal scales, as well as longer-term planning which may integrate a variety of different designs at different times. Model parameters can be easily varied, providing an ideal tool for stakeholder engagement whereby complex socio-environmental concepts and their impacts can be explored (Campbell-Arvai and Lindquist, 2021; Ferreira et al., 2020). The model represents a tool to directly include and operationalise stakeholder priorities. It allows interested parties to test different environmental scenarios and design configurations, identifying pathways which address concerns while satisfying housing needs and helping make ESS knowledge actionable (Brunet et al., 2018). While the model does not currently include monetary values, outputs can be used to inform development costs by providing information on the area and type of change. Results are communicated through a series of metrics, including a headline indicator, creating a clear presentation of change which combines a number of key indicators. This provides a useful approach to highlighting where additional interventions can be applied to improve the natural space performance of the development. By evaluating the benefits of current or potential NC, its justification for inclusion in the planning process can be derived and demonstrated, increasing the amount of blue and green space in our cities, improving urban sustainability, and enhancing quality of life.

### 5.1 Limitations and future work

While this model is designed to explore the dynamic links and feedbacks between the natural and built environment, and particularly the impacts of development on the capacity of the natural space to provide ESS, it is, like all models, an approximation of reality and is not free of biases from experts, stakeholders and modellers. The interactions between all variables have not been fully accounted for, including those between access and biodiversity, or biodiversity and hydrology. Future versions of the model will explore these additional links, along with how they interact with socio-economic variables, including income, ethnicity, age and gender. This model could be further developed to investigate the role played by blue and green space in improving physical and mental health – an increasingly important benefit on which there is limited understanding.

As discussed in previous sections, weighting can be applied to key variables to emphasise their relative importance. Data to inform these variables is typically obtained through stakeholder discussion. Due to the limited qualitative and quantitative information available from stakeholders, it was not deemed appropriate to assign differential weighting to all key variables, however, this can be varied easily within the framework by the user. This model includes key study area variables as highlighted by stakeholders and expert opinion. Such data sources have well known limitations (see Vennix (1999) for further details), yet present a key component in system understanding and model development. This data has been further supported with information obtained through scientific literature, published reports and environmental data sets. The framework is designed, for those with some SD modelling experience, to be fully adaptable and extensible and has the capacity to be used in collaboration with stakeholders to explore their priorities, thus facilitating a participatory approach to urban design which can be applied across a range of settings, and spatial and temporal scales. In addition, during the validation activity, the stakeholders highlighted the usefulness of the framework, both for system understanding and information dissemination. Further research is needed to explore potential synergies between SD and other approaches to support decisionmaking, (such as Multi-Criteria Decision Analysis). However, through system understanding and the inclusion of stakeholder highlighted key management components, this framework, allows users to weigh and prioritise development and management decisions to help achieve critical socio-environmental targets.

## 6 Conclusions

In this paper we describe the development and application of a system dynamics model to quantify and evaluate the impact of urban design on the multiple benefits and co-benefits provided by UNC and associated ecosystem services. This model provides a tool to help address a significant gap in practice, policy and research by spatially and temporally integrating the human, built and natural environment systems where key links and feedbacks are considered and represented. We have demonstrated that using a SD approach enables a more holistic ESS assessment, evaluating NC performance and allowing differing scenarios for development to be explored simultaneously. Through taking a whole system approach, this model helps identify the negative impacts of development while allowing the user to propose and assess alternative solutions. Stakeholder feedback on the framework emphasised its usefulness in triggering discussion and informing decision making. We introduce a Natural Space Performance metric; a composite of the performance outputs of key variables represented in a single headline indicator showing the propagation of change. We apply the framework to a London case study, comparing different plausible alternative permutations of the development plan. Model outputs highlight where potential improvements could be made, leading to increased green space and a reduction in flood risk. This framework helps articulate and explore the many interconnected effects of development on NC over time, allowing users to weigh and prioritise decisions, helping achieve socio-environmental targets while addressing housing and natural environment requirements.

## Supplementary Material

Supplementary data to this article can be found online at https://doi.org/10.1016/j.scitotenv.2022.153673.

Appendix A. Model documentation.

## Figures and Tables

**Table 1:** Key variables used in the model during scenarios. For a full list of variables please see Appendix A.

Variable	S1: Proposed	S2: High Rise	S3: Urban Sprawl	S4: Proposed + Green Roofs	S5: Proposed + NBS for Flood risk reduction	S6: Proposed + Green Roofs & NBS for Flood risk reduction
Building Designs (see Appx. A2 for details)	A: 4 floors	A: 14 floors	A: 2 floors	A: 4 floors	A: 4 floors	A: 4 floors
	B: 4 floors	B: 14 floors	B: 2 floors	B: 4 floors	B: 4 floors	B: 4 floors
	C: 4 floors	C: 14 floors	C: 2 floors	C: 4 floors	C: 4 floors	C: 4 floors
	D: 4 floors	D: 14 floors	D: 2 floors	D: 4 floors	D: 4 floors	D: 4 floors
	E: 2 floors	E: 10 floors	E: 1 ?oor	E: 2 floors	E: 2 floors	E: 2 floors
F: 2 floors	F: 10 floors	F: 1 ?oor	F: 2 floors	F: 2 floors	F: 2 floors
Green Roof coverage Vegetation Type Curve Number (Eq. (3))	0%	0%	0%	90%	0%	90%
	Grassland: 55	Grassland: 55	Grassland: 55	Grassland: 55	Grassland: 45	Grassland: 45
	Woodland: 40	Woodland: 40	Woodland: 40	Woodland: 40	Woodland: 40	Woodland: 40
	Blue Space:	Blue Space:	Blue Space:	Blue Space:	Blue Space:	Blue Space:
	100	100	100	100	100	100
	Marsh & Wetland:	Marsh & Wetland:	Marsh & Wetland:	Marsh & Wetland:	Marsh & Wetland:	Marsh & Wetland:
	95	95	95	95	95	95
	RUL: 50	RUL: 50	RUL: 50	RUL: 50	RUL: 42	RUL: 42
	Green roofs: 55	Green roofs: 55	Green roofs: 55	Green roofs: 55	Green roofs: 55	Green roofs: 55

## Highlights

- A new system dynamics tool to assess urban development impacts on natural capital.
- The Natural Space Performance metric indicates environmental change in urban spaces.
- Our tool captures spatiotemporal urban development and environmental interactions.

## References

[R1] Askew Phil (2018). Creating value for people in thamesmead - well being and green infrastructure.

[R2] Bateman Ian J, Georgina M (2020). The natural capital framework for sustainably efficient and equitable decision making. Nature Sustainability.

[R3] Beierle Thomas C (2002). The quality of stakeholder-based decisions. Risk Anal.

[R4] Belton, Valerie, Stewart, Theodor (2002). Multiple Criteria Decision Analysis: An Integrated Approach.

[R5] van den Berg Magdalena, Wendel-Vos Wanda, van Poppel Mireille, Kemper Han, van Mechelen Willem, Maas Jolanda (2015). Health benefits of green spaces in the living environment: a systematic review of epidemiological studies. Urban For Urban Green.

[R6] Bettencourt Luis, West Geoffrey (2010). A unified theory of urban living. Nature.

[R7] Bradbury Richard B, Stuart HM, Fisher Brendan, Francine MR, Ingwall-King Lisa, Michael A, Jennifer C, Kelvin SH, Pellier Anne-Sophie, David HL, Trevelyan Rosie (2021). The economic consequences of conserving or restoring sites for nature. Nature Sustainability.

[R8] Brunet Lucas, Tuomisaari Johanna, Lavorel Sandra, Crouzat Emilie, Bierry Adeline, Peltola Taru, Arpin Isabelle (2018). Actionable knowledge for land use planning: making ecosystem services operational. Land Use Policy.

[R9] Calliari Elisa, Staccione Andrea, Mysiak Jaroslav (2019). An assessment framework for climate-proof nature-based solutions. Sci Total Environ.

[R10] Campbell-Arvai Victoria, Lindquist Mark (2021). From the ground up: using structured community engagement to identify objectives for urban green infrastructure planning. Urban For Urban Green.

[R11] Cherry B, Pevsner N (1983). The Buildings of England. South.

[R12] Cohen-Sacham E, Walters G, Janzen C, Maginnis S (2016). Nature-based Solutions to Address Global Societal Challenges.

[R13] Coletta Virginia Rosa, Pagano Alessandro, Pluchinotta Irene, Fratino Umberto, Scrieciu Albert, Nanu Florentina, Giordano Raffaele (2020). Causal loop diagrams for supporting nature based solutions participatory design and performance assessment. J Environ Manag.

[R14] Coalition, Natural Capitalcollab (2016). Natural Capital Protocol.

[R15] Costanza Robert, de Groot Rudolf, Sutton Paul, van der Ploeg Sander, Sharolyn J, Kubiszewski Ida, Farber Stephen, Kerry Turner R (2014). Changes in the global value of ecosystem services. Glob Environ Chang.

[R16] Dasgupta P (2021). The Economics of Biodiversity: The Dasgupta Review.

[R17] Department for Environment Food and Rural Affairs (2020). Enabling a Natural Capital Approach (ENCA).

[R18] Deshmukh Dhananjay Suresh, Chaube Umesh Chandra, Hailu Ambaye Ekube, Aberra Dida, Tegene Melaku (2013). Estimation and comparision of curve numbers based on dynamic land use land cover change, observed rainfall-runoff data and land slope. J Hydrol.

[R19] Diamond Jared (1997). Guns, Germs, and Steel.

[R20] Dick Jan, Miller James D, Carruthers-Jones Jonathan, Dobel Anne J, Carver Steve, Garbutt Angus, Hester Alison, Hails Rosie, Magreehan Victoria, Quinn Melina (2019). How are nature based solutions contributing to priority societal challenges surrounding human well-being in the United Kingdom: a systematic map protocol. Environ Evid.

[R21] Eker Sibel, Zimmermann Nici, Carnohan Shane, Davies Mike (2018). Participatory system dynamics modelling for housing, energy and wellbeing interactions. Build Res Inf.

[R22] European Commission (2019). Guidance on a Strategic Framework for Further Supporting the Deployment of EU-level Green and Blue Infrastructure.

[R23] European Commission (EC) (2015). Final Report of the Horizon 2020 Expert Group on’Nature-Based Solutions and Re-Naturing Cities.

[R24] Ferreira Vera, Barreira Ana Paula, Loures Luís, Antunes Dulce, Panagopoulos Thomas (2020). Stakeholders’ engagement on nature-based solutions: a systematic literature review. Sustainability (Switzerland).

[R25] Fiori A, Volpi E (2020). On the effectiveness of LID infrastructures for the attenuation of urban flooding at the catchment scale. Water Resour Res.

[R26] Fisher Brendan, Kerry Turner R, Morling Paul (2009). Defining and classifying ecosystem services for decision making. Ecol Econ.

[R27] Fisher Dan, Blackstock Kirsty, Irvine Katherine (2020). ‘It’s on the “nice to have” pile’: potential principles to improve the implementation of socially inclusive green infrastructure. Ambio.

[R28] Ford Pauline, Baikie Ken (2018). Thamesmead: kickstarting the transformation of a stalled new town. Geography.

[R29] Frantzeskaki Niki (2019). Seven lessons for planning nature-based solutions in cities. Environ Sci Policy.

[R30] Gascon Mireia, Triguero-Mas Margarita, Martínez David, Dadvand Payam, Rojas-Rueda David, Plasència Antoni, Mark J (2016). Residential green spaces and mortality: a systematic review. Environ Int.

[R31] Giordano R, Pluchinotta I, Pagano A, Scrieciu A, Nanu F (2019). Enhancing Nature-based Solutions Acceptance through Stakeholders’ Engagement in Co-benefits Identification and Trade-offs Analysis.

[R32] Gokhelashvili Nino (2015). The role of the public in environmental decision-making. Am J Environ Protect.

[R33] Guerry Anne D, Polasky Stephen, Lubchenco Jane, Chaplin-Kramer Rebecca, Daily Gretchen C, Griffin Robert, Ruckelshaus Mary, Bateman Ian J, Duraiappah Anantha, Elmqvist Thomas, Feldman Marcus W (2015). Natural capital and ecosystem services informing decisions: from promise to practice.

[R34] Gutman Pablo (2007). Ecosystem services: foundations for a new rural-urban compact. Ecol Econ.

[R35] Haase Dagmar, Larondelle Neele, Andersson Erik, Artmann Martina, Borgström Sara, Breuste Jürgen, Gomez-Baggethun Erik, Gren Åsa, Hamstead Zoé, Hansen Rieke, Kabisch Nadja (2014). A quantitative review of urban ecosystem service assessments: concepts, models, and implementation. Ambio.

[R36] Hall Jim W, Justin J, Adrian J, Robert J (2013). Systems-of-systems analysis of national infrastructure. Proc Inst Civ Eng Eng Sustain.

[R37] Hamel P, Guerry AD, Polasky S, Han B, Douglass JA, Hamann M, Janke B, Kuiper JJ, Levrel H, Liu H, Lonsdorf E (2021). Mapping the benefits of nature in cities with the InVEST software. Npj Urban Sustain.

[R38] Holzinger O, Sadler J, Scott A, Grayson N, Marsh A (2019). NCPT – managing environmental gains and losses. 2019. “NCPT - mainstreaming green infrastructure in the planning system”. Town and Country Planning.

[R39] Horton Bruce, Digman Christopher, Ashley Richard, McMullan Jordan (2019). W047b B£ST Guidance - Guidance to Assess the Benefits of Blue and Green Infrastructure Using B£ST.

[R40] Jansson Åsa (2013). Reaching for a sustainable, resilient urban future using the lens of ecosystem services. Ecol Econ.

[R41] Jerome Gemma, Sinnett Danielle, Burgess Sarah, Calvert Thomas, Mortlock Roger (2019). A framework for assessing the quality of green infrastructure in the built environment in the UK.

[R42] Klain Sarah C, Kai MA (2012). Navigating coastal values: participatory mapping of ecosystem services for spatial planning. Ecol Econ.

[R43] Li Yafei, Fowler Hayley J, Argüeso Daniel, Blenkinsop Stephen, Jason P, Lenderink Geert, Yan Xiaodong, Selma B, Lewis Elizabeth, Feng Xiao (2020). Strong intensification of hourly rainfall extremes by urbanization. Geophys Res Lett.

[R44] Luederitz Christopher, Brink Ebba, Gralla Fabienne, Hermelingmeier Verena, Meyer Moritz, Niven Lisa, Panzer Lars, Partelow Stefan, Rau Anna Lena, Sasaki Ryuei, David J (2015). A review of urban ecosystem services: six key challenges for future research. Ecosyst Serv.

[R45] Mardani Abbas, Jusoh Ahmad, Not Khalil MD, Khalifah Zainab, Zakwan Norhayati, Valipour Alireza (2015). Multiple criteria decision-making techniques and their applications - a review of the literature from 2000 to 2014. Econ Res.

[R46] Martín Gómez, Eulalia Raffaele Giordano, Pagano Alessandro, van der Keur Peter, Costa María Máñez (2020). Using a system thinking approach to assess the contribution of nature based solutions to sustainable development goals. Sci Total Environ.

[R47] Matthews Tony, Lo Alex Y, Jason A (2015). Reconceptualizing green infrastructure for climate change adaptation: barriers to adoption and drivers for uptake by spatial planners. Landsc Urban Plan.

[R48] Mayor of London (2020). London Green Spaces Commission Report.

[R49] McGranahan G, Marcotullio P, Bai X, Balk D, Braga T, Douglas I, Elmqvist T, Rees W, Satterthwaite D, Songsore J, Zlotnik H (2005). Ecosystems and Human Well-being: Current State and Trends.

[R50] Meadows DH, Wright D (2008). Thinking in Systems: A Primer.

[R51] Mell Ian C, Henneberry John, Hehl-Lange Sigrid, Keskin Berna (2013). Promoting urban greening: valuing the development of green infrastructure investments in the urban core of Manchester, UK. Urban For Urban Green.

[R52] England Natural (2018). Monitor of Engagement with the Natural Environment. The National Survey on People and the Natural Environment.

[R53] EnglandCrosher Natural, Ian Gold, Susannah Heaver, Max Heydon, Matt Moore, Lauren Panks, Stephen Scott, Sarah Stone, Dave White Nick (2019). The Biodiversity Metric 2.0: Auditing and Accounting for Biodiversity Value. User Guide.

[R54] Notte La, Amato Dalia D, Mäkinen Hanna, Luisa Maria, Liquete Camino, Egoh Benis, Geneletti Davide, Neville D (2017). Ecosystem services classification: a systems ecology perspective of the Cascade framework. Ecol Indic.

[R55] Office for National Statistics (2019). Urban Green Spaces Raise Nearby House Prices by an Average of £2,500 - Office for National Statistics.

[R56] Office of National Statistics and Public Health England (2020). Improving Access to Greenspace. A New Review for 2020.

[R57] Ostanello A, Tsoukiàs A (1993). An explicative model of ‘public’ interorganizational interactions. Eur J Oper Res.

[R58] Pagano Alessandro, Pluchinotta Irene, Pengal Polona, Cokan Blaž, Giordano Raffaele (2019). Engaging stakeholders in the assessment of NBS effectiveness in flood risk reduction: a participatory system dynamics model for benefits and co-benefits evaluation. Sci Total Environ.

[R59] Peabody (2019). Peabody’s Plan for Thamesmead 2018 - 2023.

[R60] Pluchinotta Irene, Kazakçi Akin O, Giordano Raffaele, Tsoukiàs Alexis (2019). Design theory for generating alternatives in public decision making processes. Group Decis Negot.

[R61] Pluchinotta Irene, Pagano Alessandro, Vilcan Tudorel, Ahilan Sangaralingam, Kapetas Leon, Maskrey Shaun, Krivtsov Vladimir, Thorne Colin, O’Donnell Emily (2021a). A participatory system dynamics model to investigate sustainable urban water management in Ebbsfleet Garden City. Sustain Cities Soc.

[R62] Pluchinotta Irene, Salvia Giuseppe, Zimmermann Nici (2021b). The importance of eliciting stakeholders’ system boundary perceptions for problem structuring and decision-making. Eur J Oper Res.

[R63] Puchol-Salort Pepe, O’Keeffe Jimmy, van Reeuwijk Maarten, Mijic Ana (2021). An urban planning sustainability framework: systems approach to blue green urban design. Sustain Cities Soc.

[R64] Raymond Christopher M, Pam Berry, Breil Margaretha, Nita Mihai R, Kabisch Nadja, de Bel Mark, Enzi Vera, Frantzeskaki Niki, Geneletti Davide, Cardinaletti Marco, Lovinger Leor (2017). An Impact Evaluation Framework to Support Planning and Evaluation of Nature-Based Solutions Projects.

[R65] Reed Mark S, Graves Anil, Dandy Norman, Posthumus Helena, Hubacek Klaus, Morris Joe, Prell Christina, Claire H, Stringer Lindsay C (2009). Who’s in and why? A typology of stakeholder analysis methods for natural resource management. J Environ Manag.

[R66] Seddon Nathalie, Chausson Alexandre, Berry Pam, Girardin Cécile AJ, Smith Alison, Turner Beth (2020). Understanding the value and limits of nature-based solutions to climate change and other global challenges. Philos Trans R Soc B.

[R67] Seppelt Ralf, Dormann Carsten F, Eppink Florian V, Lautenbach Sven, Schmidt Stefan (2011). A quantitative review of ecosystem service studies: approaches, shortcomings and the road ahead. J Appl Ecol.

[R68] Sterman JD (2000). Systems thinking and modeling for a complex world.

[R69] UN (1992).

[R70] UNECE (1998). Convention on Access to Information, Public Participation in Decision Making and Access to Justice in Environmental Matters.

[R71] United Nations (2018). World Urbanization Prospects 2018.

[R72] USDA (2004). Hydrologic Soil-cover Complexes Part 630 Hydrology National Engineering Handbook Chapter.

[R73] Van Mullem JA (1989). Runoff and peak discharges using green-ampt infiltration model. J Hydrol Eng.

[R74] Vennix Jac AM (1999). Group model-building: tackling messy problems. Syst Dyn Rev.

[R75] Vennix Jac AM, Akkermans Henk A, Etiënne AJA (1996). Group model-building to facilitate organizational change: an exploratory study. Syst Dyn Rev.

[R76] Vivid Economics (2018). 30-year green infrastructure strategy for Thamesmead. Commissioned by Peabody and Landscape and Green Infrastructure Strategy for Thamesmead. Vivid Economics.

[R77] Whitford V, Ennos AR, Handley JF (2001). ‘City form and natural process’ - indicators for the ecological performance of urban areas and their application to Merseyside, UK. Landsc Urban Plan.

[R78] Whyte Jennifer, Mijic Ana, Myers Rupert J, Angeloudis Panagiotis, Alexandre Michel, Marc EJ, Ochieng Washington (2020). A research agenda on systems approaches to infrastructure. Civ Eng Environ Syst.

[R79] Yun Seong Do, Hutniczak Barbara, Abbott Joshua K, Eli P (2017). Ecosystem-based management and the wealth of ecosystems. Proc Natl Acad Sci U S A.

