# Supplementary material for: Evaluating natural capital performance of urban development through system dynamics: A case study from London
Source: Sci Total Environ. Author manuscript; Available in PMC 2024 Nov 16. (PMC7616819; doi:10.1016/j.scitotenv.2022.153673)
Supplement: Appendix A. Model documentation. [file EMS199999-supplement-Appendix_A__Model_documentation_.docx]

|  | | | | |
| --- | --- | --- | --- | --- |
| **Appendix A: Model Documentation**  This section describes in detail the parameterisation of the System Dynamics model. The model is constructed using Vensim PLE simulation software (Ventana Systems 2016) which is free to download. Please contact the authors for a free copy of the model. | | | | |
| **Housing development** | | | | |
| **Variable** | **Description** | **Equation or value** | **Initial value** | **Data Source/Comments** |
| Time | Time frame of model run in years | 30 years | - | Projected time frame of the development project |
| PROPOSED POPULATION FROM NEW DEVELOPMENT (ND) | Time series data set representing the expected increase in population over time | 0*PULSE(0,10)+6210*PULSE(10,1)+6210*PULSE(15,1)+4658*PULSE(20,1)+4658*PULSE(25,1)+4658*PULSE(25,1)+4658*PULSE(30,1) | - | Based on Peabody (2019). Total expected population over 30 years = 31500 people |
| Population in type *[type]* accommodation (6 variables, *[type]* from A through F) | Total number of people in housing unit of category *[type]* | PROPOSED POPULATION FROM NEW DEVELOPMENT (ND)*{TYPE *[type]* %} | - | Based on Peabody (2019). |
| TYPE *[type]* % (6 variables *[type]* from A through F) | The percentage of people in housing category *[type]* | *[type = A] 35; [B] 25; [C] 20; [D] 10; [E] 5; [F] 5* | - | Based on Peabody (2019). |
| Number of housing units constructed per stage | Time series data set showing the proposed increase in housing over time as a percentage of the total amount | 2300*PULSE(10,1)+2300*PULSE(15,1)+1725*PULSE(20,1)+ 1725*PULSE(25,1)+1725*PULSE(25,1)+1725*PULSE(30,1) | - | Based on Peabody (2019). |
| Number of type *[type]* housing units (6 variables, *[type]* from A through F) | The number of housing units of type *[type]* planned for the development | % TYPE *[type]* UNITS *number of housing units constructed per stage | - | Based on Peabody (2019). |
| % TYPE *[type]* UNITS (6 variables, *[type]* from A through F) | Percentage of total number of units of type *[type]* | *[type = A]* 35; *[B]* 25; *[C]* 20; *[D]* 10; *[E]* 5; *[F]* 5 | - | Based on Peabody (2019). |
| type *[type]* area (6 variables, *[type]* from A through F) | Base value of area (footprint) of a housing unit of type *[type]* | *[type = A]* 0.0061 ha; *[B]* 0.005 ha; *[C]* 0.0037 ha; *[D]* 0.0072 ha; *[E]* 0.0088 ha; *[F]* 0.01 ha | - |  |
| Building-up factor *[type]* (6 variables, *[type]* from A through F) | Factor expressing reduction in area footprint of a housing unit of type *[type]* due to allowing for more floors and higher building. | *[type = all types]* 1 | 1 | Set by user / depends on scenario. |
| Change in housing area | This variable describes the changing land area required for housing | Sum of “total type *[type]* area” for *[type] = A through F* | 0 ha | Value calculated within model from figures obtained from Peabody (2019). |
| DL initial | Initial stock of developable land | 33.4 ha | - | Initial areas calculated from Peabody (2019). |
| Increase in DL | The increase in study area space (developable land) set aside for housing. This changes to accommodate an increase in housing demand following a reduction in developable land below a minimum area. Each housing unit area is in ha. | destruction of GL + destruction of WL + destruction of MW + destruction of BS |  | Calculated within Model |
| Developable Land | This stock describes the amount of land within the study area which can be developed | Integral of {increase in DL - built area rate of change} | DL initial | Calculated within Model |
| Housing area needed for development | This stock keeps track of the housing area which was lined up for construction. If, for example, developable land is insufficient and needs to be created, then there will be a short delay of one time step in developing that area. This buffering stock allows for that. | Integral of { change in housing area - built area rate of change } | 0 ha | Calculated within Model |
| built area rate of change | The actual development of housing area, constrained by available developable land. Or, the amount of developable land used up as requirements for land to be developed due to housing construction requirements | MIN ( Developable Land (DL), Housing area needed for development ) / TIME STEP | - | Calculated within Model |
| Built Area Initial | The initial area which is built. | 0 ha | - | Calculated within Model |
| Built Area | Describes the total area converted to built area | Integral of {built area rate of change}Built Area Initial | Built Area Initial | Calculated within Model |
| DL expansion needed | Increase in developable land required to satisfy housing demand development | MAX( 0, built area rate of change - Developable Land ) / TIME STEP | - | Calculated within Model |
| **Natural spaces** | | | | |
| **Variable** | **Description** | **Equation or value** | **Initial value** | **Data Source/Comments** |
| *[land use type]* INITIAL (6 variables, *[land use type]* (in order) grassland (GL), woodland (WL), marsh and wetland (MW), blue space (BS), Reclaimed Undevelopable Land (RUL), Green roof space (GR)) | Initial stock in ha of land of type *[land use type]* | *[land use type = GL]* 6.3 ha; *[WL]* 17.2 ha; *[MW]* 6.15 ha; *[BS]* 5.45 ha; *[RUL]* 17.9 ha; *[GR]* 0.1ha | - | Initial areas calculated from Peabody (2019). |
| Addition of *[land use type]* (5 variables) | Addition of land of type *[land use type]* to the study area. This area should be subtracted from other land uses within the study area. Not applicable for Green Roof Space | User defined value (default 0 ha) | - | Taken from development plans and expert judgment. NOTE: Currently not used in the model set up |
| Green roof space factor | The share of newly built area that is converted to green roof or green roof equivalent e.g. partial green wall. | 0.9 | - |  |
| Addition of Green Roof Space (GR) | Addition of green roof space along with built area increases. | Housing area development * green roof space factor | - | Calculated within Model |
| *[land use type]* (6 variables) | The current area of land of type *[land use type]* within the study area | Integral of { Addition of *[land use type] - Destruction of [land use type]* } | *[land use type]* INITIAL | Calculated within Model |
| DL expansion needed after *[land use type]* exhausted (5 variables) | The residual developable land needed, as a rate, after land of type *[land use type]* is used up or has been reduced to its threshold value. The order in which land use types are converted into developable land is: grassland (GL), woodland (WL), marsh and wetland (MW), blue space (BS), Reclaimed Undevelopable Land (RUL)) | DL expansion needed after *[previous land use type in order]* exhausted - destruction of *[land use type]* | - | Calculated within Model |
| Toggle *[land use type]* destruction (4 variables) | Takes value 0 or 1, indicating whether land of type *[land use type]* can be converted into developable land | *[land use type = GL]* 1; *[WL]* 1; *[MW]* 0; *[BS]* 0 | - | User defined |
| Destruction of *[land use type]* (5 variables) | Reduction of area of land of type *[land use type]* for repurposing as a different land use. In this case to increase developable land | MIN (DL expansion needed after *[previous land use type in order]* exhausted , ( *[land use type]* - *[land use type]* threshold ) / TIME STEP) * Toggle *[land use type]* destruction | 0 ha | Calculated within Model |
| *[land use type]* threshold (5 variables) | Minimum/lower limit of area of land of type *[land use type]* | *[land use type = GL]* 1 ha; *[WL]* 1 ha; *[MW]* 0 ha; *[BS]* 0 ha; *[RUL]* 0 ha | - | Expert judgment |
| Natural area | Describes the total natural space within the study area (ha). This value includes land which has been reclaimed and converted into natural space (e.g. Landfill into nature reserve) | Grassland (GL) + Woodland (WL) + Marsh and Wetland (MW) + Blue Space (BS) + Reclaimed Undevelopable Land (RUL) + Green Roof Space (GR) | - | Calculated within Model |
| **Natural space performance** | | | | |
| **Variable** | **Description** | **Equation or value** | **Initial value** | **Data Source/Comments** |
| Distinctiveness | Parameters for biodiversity performance calculation. Identical across land use types. | 3 | - | Based on Biodiversity 2.0 approach and incorporating initial values suggested in the report.  ﻿<http://publications.naturalengland.org.uk/publication/5850908674228224> |
| Condition |  | 2 | - |  |
| Strategic Location |  | 1.1 | - |  |
| Connectivity |  | 1.1 | - |  |
| *[land use type]* biodiversity (6 variables) | A measure of biodiversity within the land use type using biodiversity metric 2.0 (ref). This is a function of area, distinctiveness, condition, strategic location and connectivity. | (( *[land use type]* *distinctiveness*condition)*(strategic location*connectivity)) | - | Calculated within Model. |
| Natural space biodiversity | Measure representing total biodiversity of all natural space, expressed in Biodiversity Units. | GS biodiversity + WL biodiversity + MW biodiversity + BS biodiversity + RUL biodiversity + GR biodiversity | - | Calculated within model |
| Biodiversity performance reference | Reference value for natural space biodiversity, equal to the value of that measure if the entire area were covered in the best performing land use type in terms of biodiversity. | Total area * MAX(GS biodiversity/Grassland (GL), WL biodiversity/Woodland (WL)), MW biodiversity/Marsh and Wetland (MW), BS biodiversity/Blue Space (BS),  RUL biodiversity/Reclaimed Undevelopable Land (RUL), GR biodiversity/Green roof space (GR)) | - | Calculated within model |
| Biodiversity performance relative | Natural space biodiversity relative to the reference value. | natural space biodiversity / biodiversity performance reference | - | Calculated within model |
| Biodiversity weight | Weight of biodiversity in the assessment of overall natural space performance. | 1 | - | Set by user |
| Proximity | Derived from distance from living space to green space:  1.0 - Green space less than 2min walk from home  0.5 - Green space approx. 20min walk from home  0.1 - Green space greater than 40 min walk from home  Identical across all land use types | 0.5 | - | Measured distance  Expert Judgment  Two thirds of green space visits are within 2 miles of home (PHE, 2020)  Average time to walk 2 miles = 40 mins  <https://assets.publishing.service.gov.uk/government/uploads/system/uploads/attachment_data/file/904439/Improving_access_to_greenspace_2020_review.pdf> |
| Transport | Presence of suitable public transport and parking facilities:  1.0 - excellent public transport and parking  0.5- Adequate useable public transport and parking  0.1 - Little to no useable public transport or parking  Identical across all land use types | 0.5 | - | Location specific and derived through user and expert judgment |
| Facilities and Amenities | Presence of seating, cafes, Toilets. This particularly applies to older groups and those with disabilities:  1.0 - excellent facilities including fully accessible seating areas, cafes, toilets  0.5 - Adequate facilities including fully accessible seating areas, cafes, toilets  0.1 - Little to no facilities  Identical across all land use types | 0.5 | - |  |
| *[land use type]* physical obstacles (6 variables) | Presence of suitable paths, wheelchair access, lighting and appropriate signage:  1.0 - Excellent fit for purpose paths, wheelchair accessible, well sit and sign posted  0.5 - Adequate wheelchair accessible, well sit and sign posted  0.1 - Few to no paths, Areas inaccessible typically due to poor maintenance  This variable is specific to land use type/land parcel (grassland, woodland…) | 0.8 for all land use types, except GR (green roof space) for which it is 0.01 | - |  |
| *[land use type]* access | A performance measure of the land of type *[land use type]* regarding its overall accessibility evaluated from different perspectives. | ( Proximity + Transport + Facilities and Amenities + *[land use type]* physical obstacles ) / 4 | - |  |
| Natural space access | Measure representing access performance of all natural space, calculated as the sumproduct of each land use type’s access performance and its area. | GS access * Grassland (GL) +  WL access * Woodland (WL) +  MW access * Marsh and Wetland (MW) +  BS access * Blue Space (BS) +  RUL access * Reclaimed Undevelopable Land (RUL) +  GR access * Green roof space (GR) | - | Calculated within model |
| Access performance reference | Reference value for natural space access performance, equal to the value of that measure if the entire area were covered in the best performing land use type in terms of access. | Total area * MAX(GS access, WL access, MW access, BS access, RUL access, GR access) | - | Calculated within model |
| Access performance relative | Natural space access relative to the reference value. | natural space access performance/access performance reference | - | Calculated within model |
| Access weight | Weight of access in the assessment of overall natural space performance. | 1 | - | Set by user |
| RAINFALL | Average annual rainfall in mm | 578 | - | Average monthly values for London  <https://en.climate-data.org/europe/united-kingdom/england/london-1/> |
| *[land use type]* CURVE NUMBER (6 variables) | CN is the curve number of the particular type of watershed, which has been determined by experiment. For impervious and water surfaces CN = 100, so S equals 0. For pervious surfaces CN < 100 so S is positive.  From Whitford et al 2001  However, the practical design values of land use types validated by experience lie in the range of 40 to 98 (Van Mullen, 1989). | *[land use type = GL]* 55; *[WL]* 40; *[MW]* 98; *[BS]* 100; *[RUL] 45*; *[GR]* 55 | - | Whitford et al 2001  Van Mullem, 1989  Expert Judgment |
| *[land use type]* storage | Hydrological storage capacity of land of type *[land use type]*. Numbers contained within the equations are based on experiment and are fitted and are therefore not considered external variables. | (25400/ *[land use type]* CURVE NUMBER)-254 | - | Whitford et al 2001  Van Mullem, 1989  Pandit and Gopalakrishnan (1996)  Expert Judgment  Calculated within Model |
| *[land use type]* runoff | Rainfall runoff of land of type *[land use type]* taking average annual rainfall into account. Numbers contained within the equations are based on experiment and are fitted and are therefore not considered external variables. | (RAINFALL-(0.2* *[land use type]* storage))^2/(RAINFALL+(0.8* *[land use type]* storage)) | - | Whitford et al 2001  Van Mullem, 1989  Pandit and Gopalakrishnan (1996)  Expert Judgment  Calculated within Model |
| *[land use type]* hydrological performance | Measure representing performance in hydrological terms, related to the runoff mitigating potential of the land of type *[land use type]* | 1-( *[land use type]* runoff / RAINFALL ) | - | Calculated within model |
| Natural space hydrological performance | Measure representing hydrological performance of all natural space, calculated as the sumproduct of each land use type’s hydrological performance and its area. | GS hydrological performance * Grassland (GL) +  WL hydrological performance * Woodland (WL) +  MW hydrological performance * Marsh and Wetland (MW) +  BS hydrological performance * Blue Space (BS) +  RUL hydrological performance * Reclaimed Undevelopable Land (RUL) +  GR hydrological performance * Green roof space (GR) | - | Calculated within model |
| Hydrological performance reference | Reference value for natural space hydrological performance, equal to the value of that measure if the entire area were covered in the best performing land use type in terms of hydrology. | Total area * MAX(GS hydrological performance, WL hydrological performance, MW hydrological performance, BS hydrological performance, RUL hydrological performance, GR hydrological performance) | - | Calculated within model |
| Hydrological performance relative | Natural space hydrological performance relative to the reference value. | natural space hydrological performance/hydrological performance reference | - | Calculated within model |
| Hydrological weight | Weight of hydrological performance in the assessment of overall natural space performance. | 1 | - | Set by user |
| Natural space performance | Composite indicator which expresses overall performance across land use types and combining the biodiversity, hydrological and access performance measures. | ( ( access performance relative )^access weight *  ( hydrological performance relative )^hydrological weight *  ( biodiversity performance relative ) ^biodiversity weight )^( 1 / ( access weight + hydrological weight + biodiversity weight)) | - | Calculated within model |
| Natural space performance scaling parameter | Parameter indicating the value of natural space performance for which attractiveness from natural space performance reaches 50% | 0.5 | - | Modeller judgment; set to a low value to satisfy the boundary condition of zero attractiveness when natural space performance is zero, but for acceptable values of natural space performance, the attractiveness will be close to one. |
